# Role of whole grains versus fruits and vegetables in reducing subclinical inflammation and promoting gastrointestinal health in individuals affected by overweight and obesity: a randomized controlled trial

**DOI:** 10.1186/s12937-018-0381-7

**Published:** 2018-07-30

**Authors:** Julianne C. Kopf, Mallory J. Suhr, Jennifer Clarke, Seong-il Eyun, Jean-Jack M. Riethoven, Amanda E. Ramer-Tait, Devin J. Rose

**Affiliations:** 1 Department of Food Science and Technology, University of Nebraska-Lincoln, 1901 North 21st Street, Lincoln, NE 68588-6205 USA; 2 Department of Statistics, University of Nebraska-Lincoln, Lincoln, NE USA; 3 Nebraska Food for Health Center, University of Nebraska-Lincoln, 1901 North 21st Street, Lincoln, NE 68588-6205 USA; 4 Department of Life Science, Chung-Ang University, Seoul, South Korea; 5 Center for Biotechnology, University of Nebraska-Lincoln, Lincoln, NE USA; 6 Department of Agronomy & Horticulture, University of Nebraska-Lincoln, 1901 North 21st Street, Lincoln, NE 68588-6205 USA

**Keywords:** Metabolic syndrome, Gut microbiota, Interleukin-6, Lipopolysaccharide, Tumor necrosis factor-α, C-reactive protein, Short chain fatty acids

## Abstract

**Background:**

Whole grains (WG) and fruits and vegetables (FV) have been shown to reduce the risk of metabolic disease, possibly via modulation of the gut microbiota. The purpose of this study was to determine the impact of increasing intake of either WG or FV on inflammatory markers and gut microbiota composition.

**Methods:**

A randomized parallel arm feeding trial was completed on forty-nine subjects with overweight or obesity and low intakes of FV and WG. Individuals were randomized into three groups (3 servings/d provided): WG, FV, and a control (refined grains). Stool and blood samples were collected at the beginning of the study and after 6 weeks. Inflammatory markers [tumor necrosis factor-α (TNF-α), interleukin-6 (IL-6), lipopolysaccharide binding protein (LBP), and high sensitivity C-reactive protein (hs-CRP)] were measured. Stool sample analysis included short/branched chain fatty acids (S/BCFA) and microbiota composition.

**Results:**

There was a significant decrease in LBP for participants on the WG (− 0.2 μg/mL, *p* = 0.02) and FV (− 0.2 μg/mL, *p* = 0.005) diets, with no change in those on the control diet (0.1 μg/mL, *p* = 0.08). The FV diet induced a significant change in IL-6 (− 1.5 pg/mL, *p* = 0.006), but no significant change was observed for the other treatments (control, − 0.009 pg/mL, *p* = 0.99; WG, − 0.29, *p* = 0.68). The WG diet resulted in a significant decrease in TNF-α (− 3.7 pg/mL; *p* < 0.001), whereas no significant effects were found for those on the other diets (control, − 0.6 pg/mL, *p* = 0.6; FV, − 1.4 pg/mL, *p* = 0.2). The treatments induced individualized changes in microbiota composition such that treatment group differences were not identified, except for a significant increase in α-diversity in the FV group. The proportions of Clostridiales (Firmicutes phylum) at baseline were correlated with the magnitude of change in LBP during the study.

**Conclusions:**

These data demonstrate that WG and FV intake can have positive effects on metabolic health; however, different markers of inflammation were reduced on each diet suggesting that the anti-inflammatory effects were facilitated via different mechanisms. The anti-inflammatory effects were not related to changes in gut microbiota composition during the intervention, but were correlated with microbiota composition at baseline.

**Trial registration:**

ClinicalTrials.gov, NCT02602496, Nov 4, 2017.

**Electronic supplementary material:**

The online version of this article (10.1186/s12937-018-0381-7) contains supplementary material, which is available to authorized users.

## Background

Poor diet is the leading risk factor for premature death and disability in the United States (US) [1]. Poor diets lead to metabolic syndrome and its associated diseases such as heart disease and diabetes, which rank first and seventh among common causes of death, respectively [2]. The health care cost of treating these chronic diseases is in excess of $600 billion annually [3, 4]. Consequently, the government has directed considerable policy towards promoting a healthier society, especially to promote healthier eating [5].

There have been numerous human feeding trials showing that consuming fruits and vegetables (FV) or whole grains (WG) can have significant impacts on markers of metabolic syndrome [6–14]. In a typical 2000 kcal diet, the current recommendations from the US Department of Agriculture are to consume 5 servings of FV and 3 servings of WG per day [5]. Unfortunately, FV and WG intakes are typically far below recommendations. In a 2015 report 76% of the US population did not meet the recommended intake of fruit and 87% did not meet the recommended vegetable intake [15]. Over the past 5 years FV consumption has decreased by 7% due to declines in vegetables as a side dish and consumption of juice at breakfast [16]. Likewise, a 2009–2010 survey revealed that only 2.9% of children and adolescents and 7.7% of adults in the US consumed at least 3 servings/d of WG [17]. The typical American consumes just shy of 1 serving of WG per day [17].

The metabolic benefits of WG and FV are thought to be mediated, at least in part, through their interactions with the gut microbiota [18, 19]. One way that the gut microbiota may mediate the anti-inflammatory effects of WG and FV is through short chain fatty acid (SCFA) production, the major metabolic end products of dietary fiber fermentation. These acids are known to have trophic effects locally on epithelial cell functions as well as distally via circulation in blood [20]. For instance, SCFA help with maintenance of gut barrier function by increasing mucin production, inhibiting growth of enteric pathogens, and increasing nutrient absorption [21]. Distally, SCFA are signaling molecules for carbohydrate and lipid metabolism. Increased SCFA are also associated with a decreased risk for cancer and obesity [21, 22].

Human feeding trials can vary in the design of the intervention. The most aggressive design is to control every aspect of the diet [6, 7, 11, 19, 23, 24]. However, this approach is not very practical as part of a long-term dietary regimen to improve health. A less dramatic approach is to supply a test food to subjects to incorporate into their normal diet [25–30]. This approach has some limitations compared to controlling the whole diet, such as compliance, but is easier to implement. Recently, some studies have allowed subjects to choose their own test foods from a menu consisting of foods in a particular category [12, 13, 31, 32]. While these approaches also have limitations, such as the inability to attribute outcomes to a particular food or nutrient, they may impart measureable benefits while also being more practical for subjects to continue after the study. This purpose of this present study was to determine the impact of increasing intake of WG or FV against the background of a typical Western diet on inflammatory makers and gut microbiota composition in individuals affected by overweight or obesity.

## Methods

### Study design and participants

The present study was a randomized, parallel arm feeding trial conducted at the Food Innovation Center on the University of Nebraska (UNL) Innovation Campus. The UNL Institutional Review Board approved all study protocols (Approval Number: 20141214525FB). The study was registered on clinicaltrials.gov (NCT02602496).

**Fig. 1:**
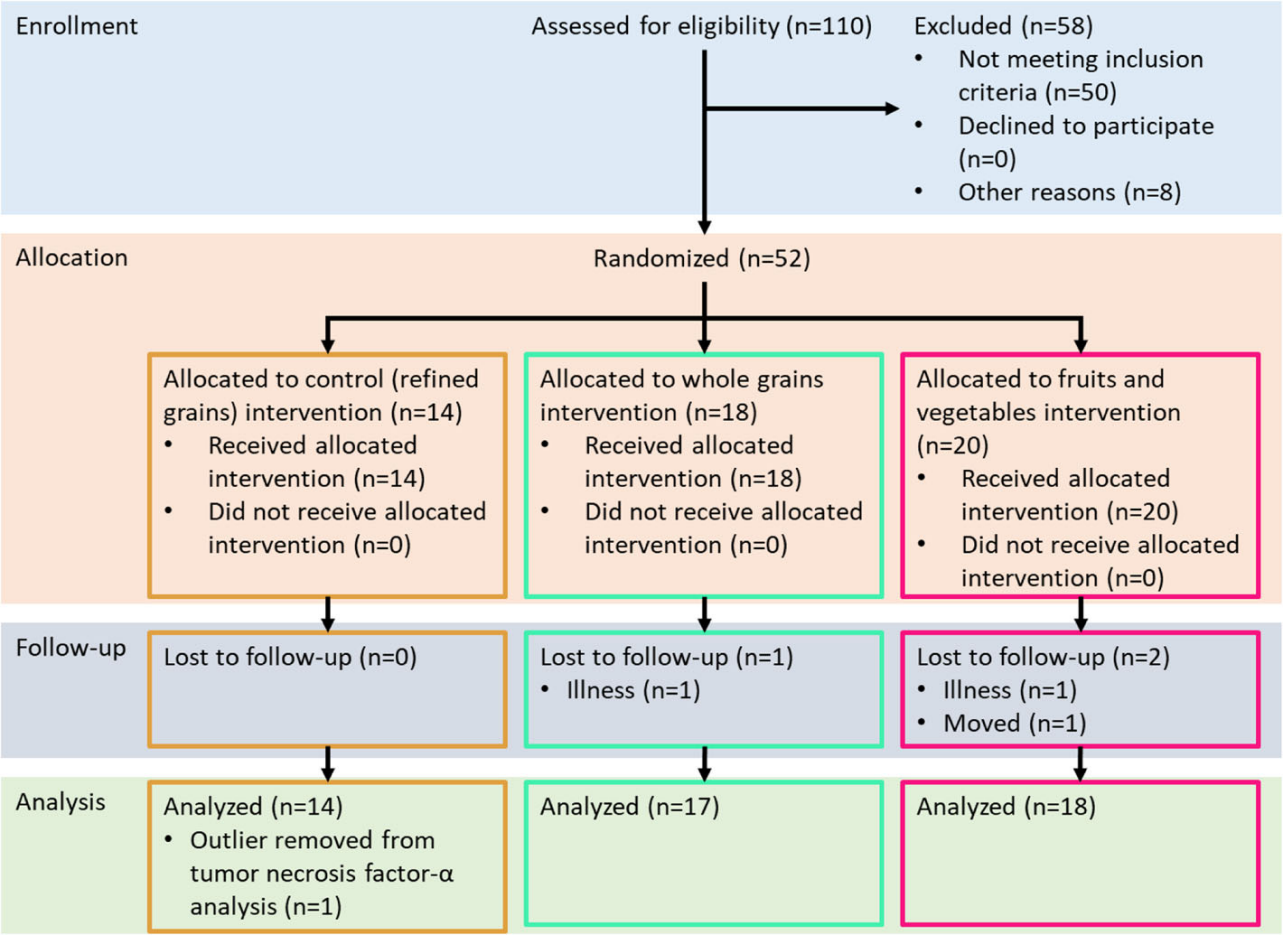
Participant flow diagram of the current study

Between August 2015 and February 2016, 110 individuals responded to flyers advertising this trial at grocery stores and University buildings and through social media outlets. Individuals were screened during the initial contact period to determine if they qualified for the study and were interested in participating. Inclusion criteria were: body mass index (BMI) > 25 kg/m^2^, no diagnosed gastrointestinal diseases, no antibiotic use for 3 months, < 1 h/week of structured exercise, and low intake of FV and WG. BMI was calculated by measuring weight and height (in light clothing without shoes). To verify a low intake of FV and WG, an online food frequency questionnaire was used [33]. The diet survey analyzed participants’ yearly diet and included questions about serving size. Responses were converted to daily intake of FV or WG using Diet*Calc software (Version 1.4.3, Bethesda, MD, USA) [34]. Sixty-one individuals were excluded due to low BMI, high intake of FV or WG, regular structured exercise, recent antibiotics use, health reasons, or scheduling conflicts. Fifty-two participants met the inclusion criteria and were enrolled in the study. Written informed consent was obtained from all subjects before being enrolled in the study. Three participants dropped out during the study due to illness or scheduling conflicts. Thus, 49 individuals completed the study (Fig. 1).

Enrolled subjects were randomized into three groups (control, WG, and FV) using an online randomization tool [35]. Participant code numbers were entered into the tool where they were randomly assigned to a group. The control group was supplied 3 servings/d of refined grain products so the study personnel could maintain contact with the participants during the study. All subjects already consumed at least 3 servings/d of these foods as part of their normal diet; therefore this treatment represented minimal to no intervention. The serving sizes supplied to each group were set according to the Nutrition Labeling and Education Act (NLEA) standards: 1 oz equivalents for WG and refined grain and 1 cup equivalents for FV [36]. Weights of 1 cup equivalents for FV were obtained using the USDA National Nutrient Database [37]. For the WG treatment, 3 servings/d brought subjects up to the minimum recommendations for US adults. For the FV group, 3 servings/d was still below the recommendations of 5 servings/d for US adults; however, we chose to keep all groups at 3 servings/d for consistency across treatment groups. Participants were instructed to incorporate the treatment foods into their normal diet.

Study participants visited the clinical facility 1 week prior to beginning the study and each week during the study. During these visits they ordered their choice of foods from a menu consisting of a list of foods within each treatment group (see Additional file 1). These foods were commonly available at the local grocery store. Subjects marked on the menu how many servings of each of the foods they wanted for the following week. Subjects could choose any combination of the foods on the list, but were required to order at least 21 and no more than 30 servings for the week.

At each of the weekly visits during the trial period, subjects turned in two diaries: one with a log of all test foods eaten during the week and the other with a record of gastrointestinal symptoms. The food diary consisted of intake of all test foods (FV, WG, and refined grain) and how many servings were consumed. Subjects recorded all test foods regardless of the treatment group to which they were assigned. The servings from the log were transferred to an online database that calculated weekly amounts of FV, total grains, WG, refined grains, and other nutrients [38]. This diary was used to assess compliance to the dietary regimens. Additionally, subjects were asked to verbally indicate compliance when they visited the facility each week.

Participants also filled out a weekly GI symptom questionnaire. Questions consisted of the frequency (number of days per week) and severity of certain symptoms, including stomach pains, heart burn, acid reflux, hunger pains, nausea, rumbling stomach, bloating, burping, flatus, constipation, diarrhea, loose stools, hard stools, urgent bowels, and feeling of incomplete bowel emptying. The severity was rated on a 0–4 scale (0 = no discomfort, 1 = slight, 2 = mild, 3 = moderate, and 4 = severe).

### Biological sample collection

Stool and blood samples were collected at the beginning of the study and after 6 weeks. The blood samples were drawn using standard venipuncture techniques by experienced phlebotomists from the University of Nebraska Medical Center. Approximately 5 mL of blood was collected into each of two tubes (367,815, Vactutainer Serum Tubes, and 367,986, Vacutainer Serum Separation Tubes, BD, Franklin Lakes, NJ USA). After collection, samples were left at room temperature for 15–30 min to allow the blood to clot. The samples were then centrifuged (2000 x g for 10 min). Supernatant (serum) was aliquoted into test tubes for storage at − 80 °C until analysis.

Stool samples were collected using a commode collection kit (02–544-208, Thermo Fischer Scientific, Waltham, MA USA). An insulated cooler with ice packs was provided to keep samples cool until they could be delivered to the research center for immediate storage at − 80 °C. The samples were obtained and frozen within 2 h of defecation.

### Biological sample analysis

TNF-α and LBP were assayed from the serum recovered from the serum tubes; IL-6 and hs-CRP were assayed from the serum recovered from the serum separation tubes. All analyses were carried out using commercial ELISA kits according to the manufacturer’s instructions (hs-CRP: HU8817; TSZ, Waltham, MA USA; TNF-α: KHC3011, Invitrogen, Frederick, MD USA; IL-6: HS600B, R&D Systems Minneapolis, MN USA; LBP: 0628D2100, Sigma Aldrich, St. Louis, MO USA).

For stool sample analysis, stool was first homogenized with ice-cold, sterile phosphate buffer (1:10 *w*/*v*). Fecal homogenates were then transferred into a 2 mL sterile bead-beating tube containing 300 mg of 0.1-mm zirconium beads, and bacterial cells were collected by centrifugation at room temperature at 8000 x g for 5 min. The supernatant was used for S/BCFA analysis and the bacterial pellet was washed an additional two times with PBS in preparation for bacterial DNA extraction.

S/BCFA analysis was performed as described [39].

DNA was extracted from the bacterial pellet using the phenol:chloroform:isoamyl alcohol with bead beating method described [14]. Following extraction, the DNA was resuspended in 0.1 mL of tris buffer (10 mM, pH 8) and frozen at − 80 °C until sequencing.

The V4-V5 region of the 16S rRNA gene was amplified by PCR using primers Meta_V3_F_Nextera: (5’-CCTACGGGAGGCAGCAG-3′) and Meta_V4_806_R: (5’-GGACTACHVGGGTWTCTAAT-3′). PCR reactions were performed using KAPA HiFidelity Hot Start Polymerase. After the first round of amplification, PCR products were diluted 1:100 and a second PCR amplification was performed on the diluted sample. Pooled, size-selected sample was denatured with NaOH, diluted with HT1 buffer (Illumina) to 8 pM, spiked with 20% PhiX, and denatured at 96 °C for 2 min immediately prior to loading. A MiSeq 600 cycle V3 kit was used to sequence the sample.

Following sequencing, base sequence quality information was confirmed by FastQC [40]. Reads with base quality scores below the minimum (30 per base) across the whole read or read length less than 30 bases were removed using Trim Galore (ver. 0.4.0) [41].

Filtered sequences were analyzed through the QIIME pipeline (ver. 1.9) [42] using scripts implemented within QIIME. Paired-end reads were merged and were clustered into operational taxonomic units (OTUs) at a sequence similarity level of 97% by UCLUST (default parameters). OTUs that had less than 10 reads mapped were removed. All raw sequences from this study were deposited in the National Center for Biotechnology Information (NCBI) Sequence Read Archive (SRA) under accession number SRP125515.

### Statistical analysis

All data with the exception of OTU comparisons were analyzed using SAS software (version 9.4, SAS Institute, Cary, NC, USA). As mentioned, 52 subjects were enrolled in the study. With this number of subjects, we estimated that we would be able to detect an effect size of 0.4 with 80% power. This was based on our published results from a whole grain barley and brown rice feeding trial, where we saw an effect size of 0.48 for change in IL-6 with the whole grain treatment [25]. Following the intervention, differences across treatment groups were assessed using ANOVA where treatment group was the main factor and BMI (at baseline), gender, and baseline outcome measurement were covariates. Changes in measured variables from baseline to the end of the study within each group were also assessed after correcting for BMI (at baseline), gender, and baseline values. Stool bacterial data was log2 transformed for analysis. Correlations were analyzed using Pearson’s method. Changes in OTU abundances were determined using DESeq2 (ver. 1.14) [43] in the R Bioconductor package (http://www.bioconductor.org) (ver. 3.1.2). *P*-values for stool bacterial data and correlations were corrected using the false discovery rate procedure. All analyses corrected for baseline concentrations, age, gender, and body mass index. One subject was excluded from the control group in the IL-6 analysis due to an outlying value (15.7 pg/mL). *P*-values for other comparisons were adjusted using Tukey’s procedure. Adjusted *p* < 0.05 was considered significant.

## Results

### Baseline characteristics

There were no significant differences among treatment groups at baseline except for age (Table 1). Participants in the WG group were significantly older than the control group. This was due to a higher proportion of subjects that were over 40 years of age (52% compared with 11% in the FV group and 0% in the control group). We are uncertain as to the cause of this significant difference as subjects were randomized into treatment groups. It is known that composition and activity of the microbiome changes as one ages [44, 45]. There were two elderly subjects (> 70 years of age) in the study: one in the WG group and the other in the FV group.

**Table 1:** Baseline characteristics of subjects that completed the study (*N* = 49)

	Treatment group			
Baseline data	Control	WG	FV	*P*-value
Subjects (N)	14	17	18	
Gender (M/F)	7/7	6/11	6/12	0.20
Age (years)	27.6 ± 5.9 b	39.2 ± 13.5 a	29.4 ± 12.8 ab	< 0.01
BMI (kg/m^2^)	30.1 ± 5.2	33.7 ± 6.3	30.3 ± 6.0	0.40
Plasma inflammatory markers				
IL-6 (pg/mL)	2.9 ± 1.5	4.4 ± 1.9	4.3 ± 2.6	0.60
TNF-α (pg/mL)	23.8 ± 5.9	26.7 ± 4.17	24.2 ± 5.2	0.11
hs-CRP (mg/mL)	0.6 ± 0.4	0.8 ± 0.6	0.7 ± 0.4	0.89
LBP (mg/L)	1.8 ± 0.3	1.9 ± 0.4	1.8 ± 0.4	0.38
Stool short chain fatty acids (mmol/g feces)				
SCFA	101 ± 71	59.9 ± 39.7	64.0 ± 36.1	0.29
Acetate	64.6 ± 38.2	42.9 ± 27.9	46.6 ± 28.0	0.43
Propionate	20.4 ± 23.0	9.74 ± 7.65	9.61 ± 5.93	0.30
Butyrate	16.3 ± 12.8	7.14 ± 5.32	7.80 ± 5.15	0.07
BCFA	2.34 ± 1.74	1.74 ± 1.30	1.52 ± 0.78	0.37
Stool microbiota composition (relative abundance, %)				
Actinobacteria	6.54 ± 4.08	5.99 ± 3.39	4.42 ± 4.32	0.36
Bacteroidetes	13.8 ± 9.7	11.2 ± 5.7	12.3 ± 8.1	0.38
Firmicutes	78.8 ± 9.2	81.0 ± 8.4	82.4 ± 9.5	0.28
Proteobacteria	0.67 ± 0.08	1.14 ± 1.92	0.43 ± 0.55	0.26
Verrucomicrobia	0.18 ± 0.42	0.62 ± 1.13	0.46 ± 0.94	0.75
Other	0.04 ± 0.11	0.07 ± 0.14	0.05 ± 0.14	0.79

Mean ± standard deviation; *WG* whole grain, *FV* fruits and vegetables, *BMI* body mass index, *IL-6* interleukin-6, *TNF* tumor necrosis factor-α, *hs-CRP* high-sensitivity C-reactive protein, *LBP* lipopolysaccharide binding protein, *SCFA* short chain fatty acids, *BCFA* branched chain fatty acids

There were a few notable non-significant observations in the baseline data concerning the microbiota (Table 1). First, the fecal butyrate concentration seemed unusually high in the control group. While not significant, it was concerning to observe these high values at baseline. The reasons for the apparently high butyrate concentration in the control group is not known. Second, we observed that the Bacteroidetes phylum appeared underrepresented and the Firmicutes phylum overrepresented in this sample population compared with several studies [11, 19, 25], although this may not be a relevant issue [46, 47]. The low abundance of Bacteroidetes could be due to the characteristics of the type of subjects recruited for this study. It could also be due to bias during fecal DNA extraction. Importantly, DNA was extracted and sequencing was performed on baseline and end of study fecal samples simultaneously. Therefore, any bias created during the analysis would likely affect all samples uniformly.

### Changes in host characteristics

Based on diet diaries, participants in the WG and FV groups were compliant with the treatment protocols (Table 2). The diaries also showed that the interventions were treatment specific; e.g., subjects in the FV group did not increase consumption of WG foods while subjects in the WG group avoided FV. Based on intake of refined grain in the control group it appeared that the subjects in the WG group replaced refined grain foods with WG foods. Oddly, despite repeated instructions, it appeared that the subjects in the FV group may not have recorded all refined grain foods consumed. Perhaps they were more concerned about making sure to record all treatment foods.

**Table 2:** Average daily intake of treatment foods recorded by subjects in diet diaries

Treatment Groups	Refined grain (oz. eq.)	Fruit (cup eq.)	Vegetables (cup eq.)	FV (cup eq.)	WG (oz. eq)
Control	7.1 ± 0.7 a	0.7 ± 0.1 b	0.3 ± 0.1 b	1 ± 0.1 b	0.7 ± 0.1 b
WG	2.7 ± 0.7 b	0.4 ± 0.1 b	0.3 ± 0.1 b	0.7 ± 0.1 b	3.4 ± 0.2 a
FV	2.4 ± 0.4 b	1.6 ± 0.1 a	1.2 ± 0.1 a	2.9 ± 0.2 a	0.9 ± 0.3 b

Mean ± standard deviation; means followed by different letters in the same column are significantly different (*p* < 0.05)

Weekly gastrointestinal symptoms were recorded to track changes throughout the study. There were no significant changes in GI symptoms throughout the study, indicating that the treatments were well-tolerated (see Additional file 2).

BMI was recorded at baseline and at the end of the study. There were no significant changes in BMI (see Additional file 3).

**Fig. 2:**
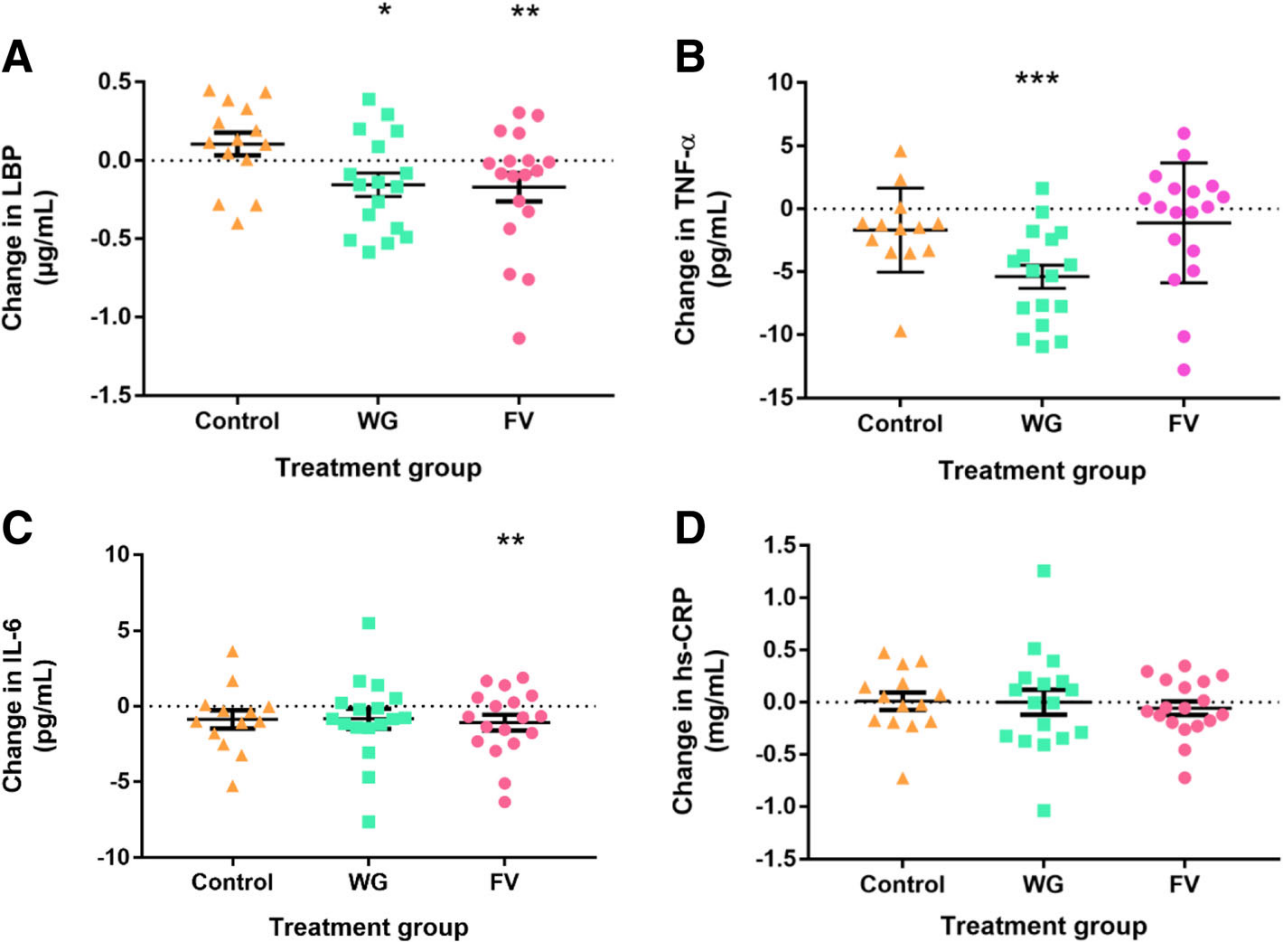
Changes in inflammatory markers during the treatment period. **a**) Lipopolysaccharide binding protein (LBP); **b**) tumor necrosis factor (TNF)-α; **c**) interleukin (IL)-6; and **d**) high sensitivity C-reactive protein (hs-CRP); * *p* < 0.05, ** *p* < 0.01, *** *p* < 0.001 for change across time = 0; all analyses corrected for baseline concentrations, age, gender, and body mass index; one subject was excluded from the control group in the IL-6 analysis due to an outlying value (15.7 pg/mL)

There were significant decreases in LBP from baseline values in participants consuming the WG and FV diets with no change for those on the control diet (Fig. 2). Additionally, the WG diet resulted in a significant decrease in TNF-α levels during the intervention, whereas no significant changes in TNF-α were found for those on the other treatment. There were no significant changes in circulating IL-6 levels for subjects receiving the control and WG treatments for IL-6, but there was a significant decrease in FV diet. There were no significant changes from baseline for hs-CRP levels for any subjects.

### Changes in microbiota characteristics

There were no significant changes in fecal short chain fatty acid (SCFA) production during the study period for any participants (see Additional file 3). The apparently high fecal butyrate in the control group at baseline appeared to remain high for the duration of the study such that no significant change over time was observed.

**Fig. 3:**
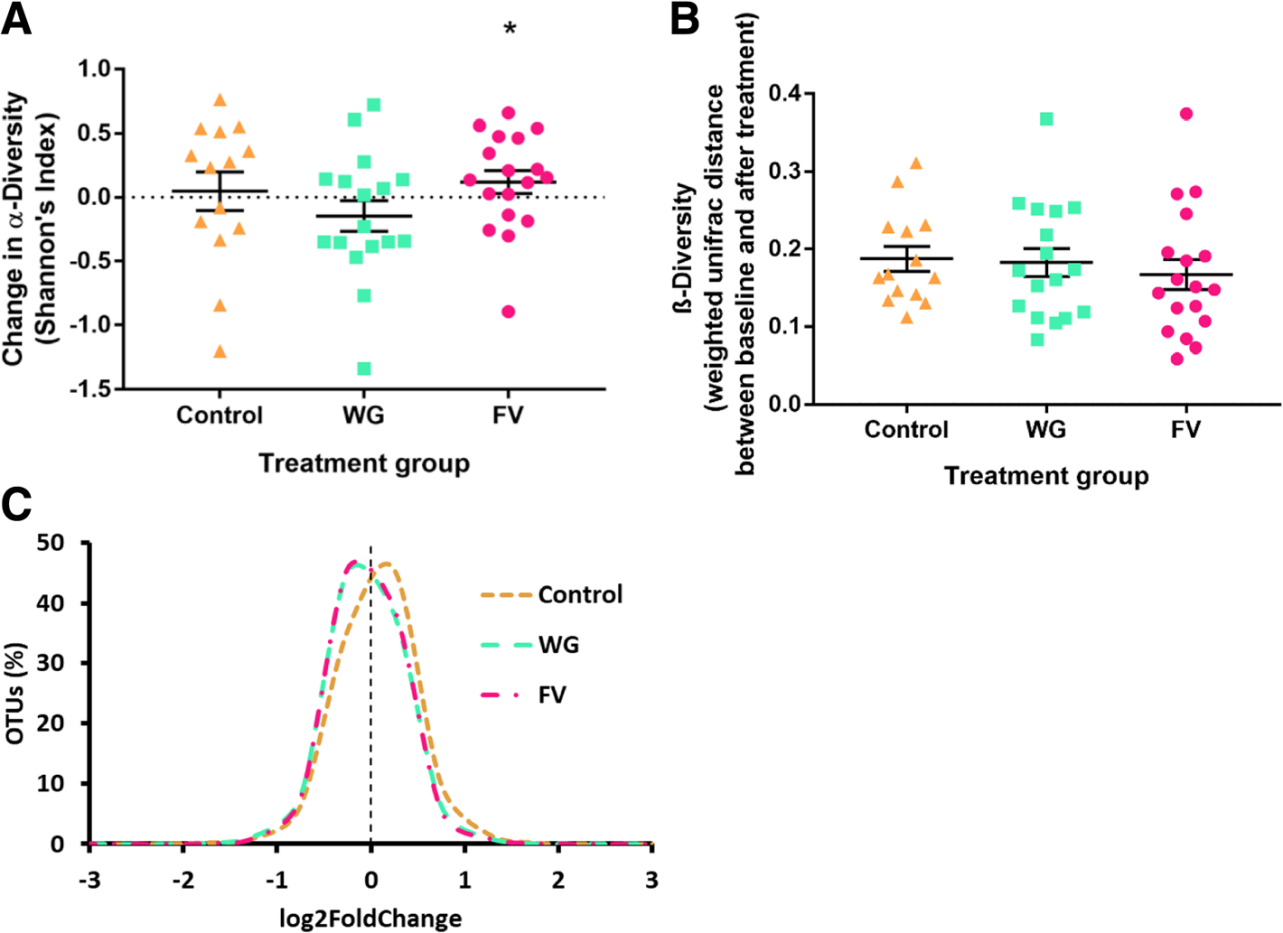
Changes in fecal microbiota during the treatment period. **a**) α-Diversity; **b**) β-diversity; and **c**) operational taxonomic unit (OTU) fold-change; * *p* < 0.05, for change across time = 0; diversity measures corrected for baseline concentrations, age, gender, and body mass index

**Fig. 4:**
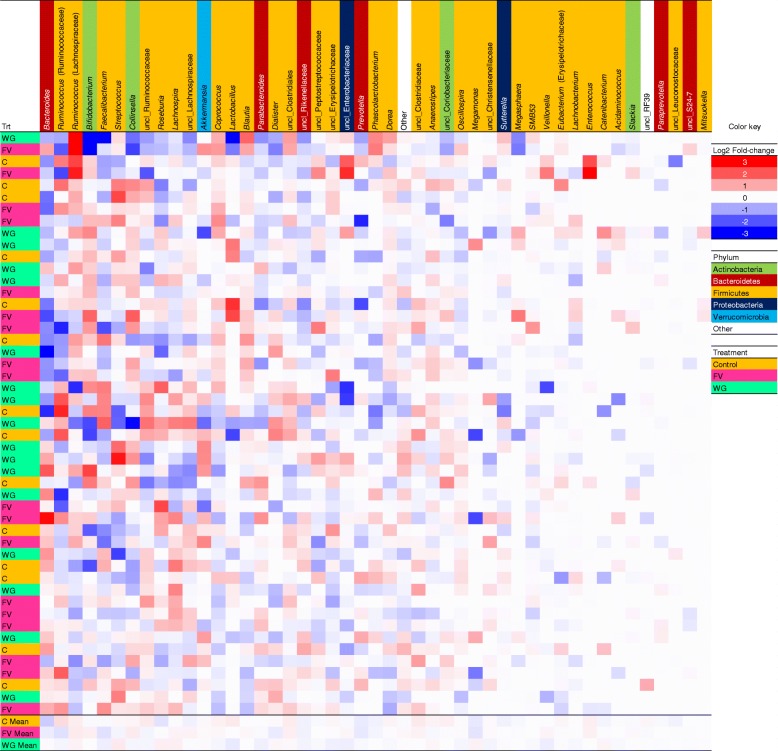
Change in dominant bacteria in fecal samples from baseline to the end of the treatment. Treatment groups are clustered using hierarchical clustering (Ward’s method); taxa are ordered by absolute change in abundance across all subjects; uncl, unclassified; C, control; FV, fruits and vegetables; WG, whole grain

The FV intervention resulted in a significant increase in α-diversity, a measure of species richness, while the other treatment groups did not show any effect (Fig. 3). β-Diversity, a measure of the change in species richness over time, was not significantly different across treatment groups. Additionally, there were no significant differences between treatment groups from baseline to the end of treatment at the OTU level. When OTUs were binned according to their relative fold change during the intervention, the WG and FV treatment groups showed a shift toward a decrease in some OTUs of < 0.5-fold while the control treatment group showed a shift toward an increase of < 0.5 fold. No significant change in bacterial genera were found, but there were observable individualized responses to the treatments (Fig. 4). Based on the cluster analysis, *Bacteroides*, *Ruminococcus* (Ruminococcaceae), *Ruminococcus* (Lachnospiraceae), *Bifidobacterium*, and *Fecalibacterium* showed the most change from baseline to the end of the study, but changes were not dependent on treatment groups.

### Association between changes in inflammatory markers and microbiota composition

**Fig. 5:**
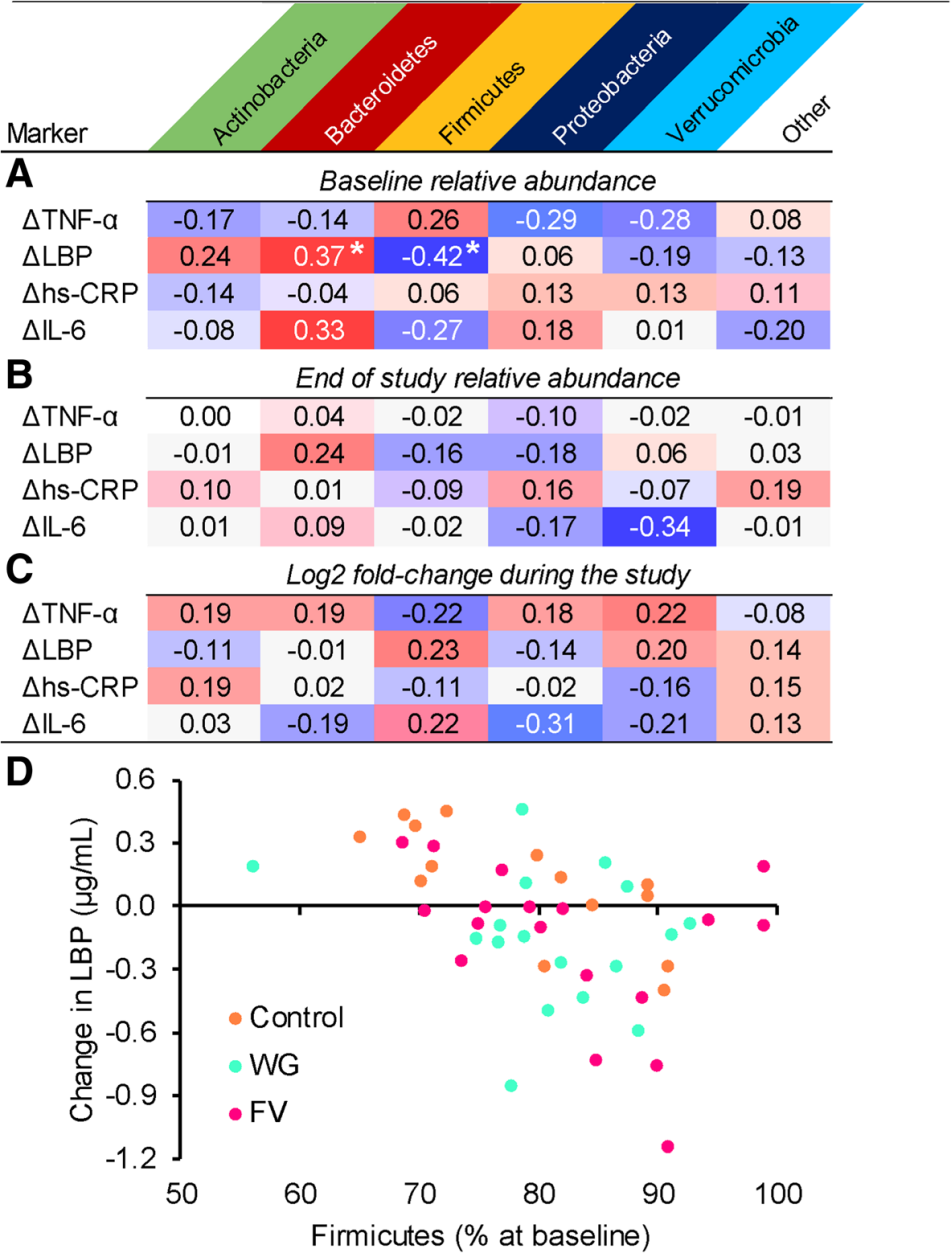
Correlations between change in plasma markers during the study and microbiota composition. **a**) Baseline abundance; **b**) end of study abundance; and **c**) log2 fold-change during the study of stool microbiota; **d**) scatterplot of baseline abundance of Firmicutes and change in lipopolysaccharide binding protein (LBP) during the study; *LBP* lipopolysaccharide binding protein, *TNF-α* tumor necrosis factor, *IL-6* interleukin-6, *hs-CRP* high sensitivity C-reactive protein, *WG* whole grain, *FV* fruits and vegetables; partial variables were treatment group, age, gender, and body mass index; *N* = 49 except IL-6 where *N* = 48; *p*-values were corrected for false discovery rate; * adjusted *p* < 0.05

Changes in inflammatory makers during the study appeared to be more correlated with baseline microbiota composition than with changes in microbiota composition during the study or composition at the end of the study (Fig. 5). Significant correlations were found between baseline microbiota composition and change in LBP during the study. In particular, individuals with higher Firmicutes and lower Bacteroidetes showed a greater decrease in LBP during the study. At deeper taxonomic levels, it appeared that this was due mostly to abundance of a few genera of the Clostridales order (see Additional file 4).

## Discussion

In the present study we aimed to evaluate the impact of increasing WG or FV intake on inflammatory makers and gut microbiota composition in individuals affected by overweight or obesity with normally low intakes of these foods. Subjects increased their intake of these foods from < 1 serving/d to 3 servings/d. Subjects did not all consume the same foods, but rather consumed their choice of foods from a particular food category. In this respect, our intervention was very modest, focusing on a potentially sustainable change in participants’ diet pattern. Anecdotally, many subjects in each group commented on how different they felt their diet was. Many commented that they “[hadn’t] eaten this healthy in years.” During the weekly visits to the study facility, several subjects in the FV group often indicated that it was very difficult to adhere to the treatment regimen. No such comments were made by individuals in the other two groups. Many participants indicated informally that they planned to continue trying to consume more FV or WG after the study period.

One limitation of this experimental design is that subjects used diet diaries to record their food intake. While diet diaries have the advantages that they can provide detailed food intake data without the constant presence of study personnel, they do have disadvantages including accidental or intentional under or over-reporting of foods consumed or portion sizes [48]. For example, our results suggested that participants in the FV group may have under-reported their intake of refined grain foods. However, the weekly visits to the clinical facility coupled with the positive biological effects observed in the treatment groups suggests that subjects generally conformed to the treatment protocols.

The WG and FV diets had significant positive impacts on inflammatory markers. The FV treatment decreased LBP and IL-6, while the WG foods decreased LBP and TNF-α. Previous studies with FV and WG have also reported reductions in inflammatory markers [6–10, 12, 24, 25], although some other studies have not shown significant differences [23, 30, 31, 49]. The differences in experimental design and treatment foods make direct comparisons among studies difficult. Lipopolysaccharide is a component of the cell walls of gram-negative bacteria; increased levels of LBP in the blood is suggestive of endotoxemia [50]. The change in LBP in both groups suggested a positive impact on gut barrier function. The decreases in either IL-6 or TNF-α suggest reductions in subclinical inflammation, which is associated with a lower risk of metabolic syndrome.

One limitation of this study is that with the variety of food options provided to subjects it was not possible to pinpoint which component of the foods were responsible for the biological effects observed. Both treatment groups were good sources of dietary fiber, although the composition of the dietary fibers within each group were very different. The major dietary fibers in WG are β-glucan, arabinoxlyan, and cellulose, while FV primarily contains pectins, xyloglucan and cellulose [51, 52]. FV also contain more soluble fiber, and WG contain more insoluble fiber [52–54]. Additionally, FV have more free polyphenols, while WG have a predominance of bound phenolics [55]. WG foods can also contribute vitamin E, and phytoserols that have been linked to lowering markers of metabolic syndrome [56]. In contrast, FV include folate, flavonoids, vitamin C, and β-carotene, which have been inversely correlated with hs-CRP and IL-6 [57].

We did not find any effects of WG or FV on hs-CRP, even though previous studies have reported significant effects [6, 9, 13, 27]. Post-hoc power analysis revealed that we had only 12% power to detect differences in hs-CRP due to our modest sample size. Therefore, even if WG or FV had significant effects on hs-CRP we would likely not be able to detect it. Notably, our study was powered to detect differences in IL-6, where we did find differences. We also found differences in LBP and TNF-α, indicating sufficient power for those inflammatory markers.

One unique aspect of the present study is the comparison between WG and FV. There are several previous studies that test WG in comparison with refined grain or high and low FV diets [6, 7, 9–13, 19, 24–28, 30–32, 49]. However, no previous studies have included both food groups in the same study. Interestingly, while both treatment groups decreased inflammatory markers, each decreased a different biomarker. These unique changes suggest that the beneficial effects of WG and FV on inflammation may be mediated via different mechanisms. Furthermore, perhaps consuming both FV and WG together could have a synergistic effect to help lower inflammation. There are some diets that combine, among other dietary recommendations, WG and FV to improve health (e.g., DASH). For instance, a clinical trial of the DASH diet on type 2 diabetic patients showed a significant reduction in CRP [58, 59], although other similar feeding trials have not shown reductions in CRP or markers of inflammation [60]. In a study where participants followed a Nordic diet, a reduction in expression of genes related to inflammation in subcutaneous adipose tissue was reported [61].

The significant decrease in LBP during the interventions suggested a link between the changes in inflammatory state and the gut microbiota. However, save for an increase in α-diversity in the FV group, we found no significant changes in gut microbiota composition by treatment group during the intervention. Rather, found that gut microbiota composition at baseline was more related to changes in LBP than changes in the gut microbiota during the intervention. Others have also found that baseline microbiota composition is associated with changes in outcomes during an intervention trial. Korpela et al. [18] reported that baseline microbiota composition had the greatest ability to predict changes in host cholesterol levels using data from three independent intervention studies. Kovatcheva-Datchary et al. [28] found that baseline abundances of *Prevotella* were associated with host improvements in glucose tolerance. In our study, it appeared that certain members of the Clostridiales order were perhaps involved in the reduction in LBP following either a WG or FV intervention. The Clostridiales order contains a number of important gut microbiota families, such as Lachnospiraceae and Clostridiaceae, which contain bacteria that are important in the degradation of complex carbohydrates [62, 63].

The increase in α-diversity during the FV diet may have been due to the introduction of the wide variety of new dietary fibers to subjects’ diets. In contrast, the WG diet was mainly composed of wheat-based products which contain similar dietary fibers to the refined grain products that subjects consumed as part of their habitual diet prior to the study.

There have been several studies that report changes in the gut microbiota following a WG intervention. Most studies report only modest to no changes in gut microbiota composition in accordance with our study [10, 11, 13, 26, 32, 49]. Others have reported more substantial changes, such as increases in beneficial bacteria and even phylum-level changes [14, 64]. The differences in gut microbiota changes may be due to differences in the types, forms, or quantities of WG consumed in each of the studies. Notably, among those microbial taxa that changed the most during the study, our power to detect significant shifts only ranged from 25 to 50%. Therefore, the treatments may have induced shifts in the microbiota that we were unable to detect due to insufficient sample size. Notably, our study was powered to detect differences in inflammatory markers (IL-6) and not microbiota changes.

Very few studies have reported on the changes in gut microbiota composition following a FV intervention. Li et al. [19] reported shifts in the gut microbiota structure following a cruciferous vegetable intervention, but the shifts were dependent on individuals. Despite the individualized responses, four taxa were associated with the cruciferous vegetable diet: *Eubacterium hallii*, *Phascolarctobacterium faecium*, *Alistipes putredinis*, and *Eggerthella* spp. In another study also feeding cruciferous vegetables, the authors reported significant reductions in sulfate-reducing bacteria [65]. This is desirable, since sulfate is reduced to hydrogen sulfide by sulfate-reducing bacteria, which has been associated with several GI disorders [66, 67].

This present study did not show significant changes in fecal SCFA. This has also been reported by others: a three-week treatment of WG cereal showed no significant difference between whole grain treatment and the control in SCFA production [64]. SCFA are rapidly absorbed from the gut, which could be why many studies, including our study, show no significant changes in SCFA production [68]. However, when using a more dramatic treatment of a 12 week diet rich in WG foods, the authors reported increased plasma propionate concentrations [11].

## Conclusions

FV and WG interventions significantly and uniquely reduced biomarkers of inflammation. The FV treatment decreased circulating IL-6 and LBP and while the WG treatment decreased TNF-α and LBP. Both treatments had individualized effects on the gut microbiota, with a significant increase in α-diversity in the FV treatment. These data support the positive impact that WG and FV intake can have on metabolic health in individuals affected by overweight or obesity with normally low intake of WG and FV.

## Additional files

Additional file 1: Treatment foods supplied to subjects. (XLSX 10 kb)

Additional file 2: Gastrointestinal symptom questionnaire results. (XLSX 10 kb)

Additional file 3: Characteristics of subjects that did not show significant changes during the study. (XLSX 10 kb)

Additional file 4: Correlations between change in plasma markers during the study and microbiota composition. (XLSX 16 kb)

## Abbreviations

BCFA: Branched chain fatty acids
Hs-CRP: High sensitive C-reactive protein
IL-6: Interleukin 6
LBP: Lipopolysaccharide binding protein
SCFA: Short chain fatty acids
TNF-α: Tumor necrosis factor alpha
